# Cell-Seeded Acellular Artery for Reconstruction of Long Urethral Defects in a Canine Model

**DOI:** 10.1155/2021/8854479

**Published:** 2021-06-04

**Authors:** Hao Zhong, Yamei Shen, Danhui Zhao, Guoqiang Yan, Chengai Wu, Guanglin Huang, Zhizhong Liu, Jianpo Zhai, Qin Han

**Affiliations:** ^1^Department of Urology, Inner Mongolia Baogang Hospital, Baotou 014010, China; ^2^Institute of Basic Medical Sciences Chinese Academy of Medical Sciences, School of Basic Medicine Peking Union Medical College, Peking Union Medical College Hospital, Center of Excellence in Tissue Engineering Chinese Academy of Medical Sciences, Beijing Key Laboratory (No.BZO381), Beijing 100010, China; ^3^Beijing Research Institute of Traumatology and Orthopaedics, Beijing 100035, China; ^4^Department of Urology, Beijing Jishuitan Hospital, Beijing 100096, China

## Abstract

The management of urethral stricture remains a major therapeutic challenge in clinics. Herein, we explored the feasibility of reconstructing a relatively long segment of the urethra by the cell-seeded acellular artery in a canine model. The acellular arterial matrix was obtained from the excised carotid artery of donor dogs. Autologous adipose-derived stem cells (ADSCs) from 6 male dogs were grown and seeded onto the premade acellular arterial matrix. A 3 cm long segment of the urethra was resected in 12 male dogs. Urethroplasty was performed with the acellular arterial matrix seeded with ADSCs in 6 animals and without cells in 6. Serial urethrography was performed at 1 and 3 months postoperatively. Wide urethral calibers without any signs of strictures were confirmed in all 6 animals in the experimental group. In contrast, urethral stricture was demonstrated in 3 animals in the control group. The graft was highly epithelialized and smooth in the experimental group, while graft contracture and scar formation were showed in the control group. Histologic analysis of the cell-seeded arterial matrix at 1 month confirmed the presence of multilayered urothelium and muscle. The levels of tissue formation developed over time with a progressive increase in muscle content. In contrast, extensive fibrosis and sparse smooth muscle were seen in animals treated with matrix without ADSCs. This study provides preclinical evidence that the ADSC-seeded arterial matrix can be used as a tubularized scaffold in the reconstruction of 3 cm long urethral defect in a male canine model. The ADSC-seeded arterial matrix remodels and regenerates normal-appearing urethral tissue layers over time.

## 1. Introduction

The management of urethral stricture remains a major therapeutic challenge in clinics. In men, iatrogenic injury is the most common cause of urethral stricture followed by idiopathy, trauma, and inflammation [1–4]. Systemic diseases, such as lichen sclerosus, can also lead to urethral strictures [5, 6]. Scarring of the urethral tissue is the causative process leading to the replacement of the vascular tissue of the corpus spongiosum, which leads to ischemic spongiofibrosis of the urethra [7, 8]. Urethral strictures present a serious health condition that significantly impairs quality of life and may lead to the failure of vital organs if left untreated [9].

Repair of the anterior urethra is one of the most demanding surgical problems in urology. Several autologous tissues have been proposed for urethroplasty as free grafts or flaps, with the success rates reported between 75 and 85%. Nevertheless, it is not without complications, such as donor site morbidity and prolonged hospitalization [10–13]. Regenerative medicine and tissue engineering studies have led to the development of novel biomaterials for urethral repair [14–17]. Choosing the right cell type together with a supporting matrix is a crucial step in urethral tissue engineering [18]. The development of an acellular matrix by tissue engineering represents a remarkable stage in the field of reconstructive surgery [19]. Several studies reported successful results when acellular matrixes were used to replace partial urethral defect in an on-lay fashion. Sievert et al. used a tubular acellular matrix to replace a circumferentially excised segment of a rabbit urethra with 0.8–1.1 cm long and reported satisfactory results [20]. However, it is uncertain whether the acellular artery could serve as a graft material for urethral stricture diseases. In the present study, we investigated the feasibility of using acellular arteries to engineer a long urethral segment in canines.

## 2. Materials and Methods

This study was conducted with the approval of the Ethics Committee of Beijing Jishuitan Hospital. All animal experiments complied with the ARRIVE guidelines and were carried out in accordance with the U.K. Animals (Scientific Procedures) Act, 1986 and associated guidelines. A total of 12 male Beagle dogs with an average age of 1 y and average weight of 8.7 kg, purchased from Beijing Marshall Biotechnology Co. Ltd., were included in the study. The acellular arterial matrix was obtained from the excised carotid artery of male donor dogs that were not included in the study. Autologous adipose-derived stem cells (ADSCs) from 6 male dogs were grown and seeded onto a premade acellular arterial matrix (3 cm in length). The long urethral segment was resected in the 12 male dogs. Urethroplasty was performed with the acellular arterial matrix seeded with ADSCs in 6 animals and without cells in the other 6. Serial urethrography was performed at 1 and 3 months postoperatively. The animals were sacrificed for analysis at predetermined time points (three animals in each group at 1 month and 3 months, respectively).

### 2.1. Preparation of the Arterial Matrix

Carotid arteries were obtained by surgical resection from the donor animal. The carotid arteries were rinsed with phosphate-buffered saline at 4°C, followed by treatment with 0.03% trypsin for 2 h at room temperature. The matrix was then treated with a solution of Triton X-100 (0.5%) and ammonium hydroxide (0.05%) in distilled water for 24 h at 4°C to dehydrate the cells. Finally, 10 mmol/L Tris-HCl washing solution containing 50 U/mL DNase I and 1 U/mL RNase A was added and washed at room temperature for 24 hours with shaking to digest the nucleus and remove the DNA and RNA components in the carotid artery. The tissue was washed, frozen, and lyophilized, and after sterilization with ethylene oxide, it was sealed and stored at 4°C.

### 2.2. Cell Isolation and Expansion

The animals were sedated with acepromazine (0.05 mg/kg intramuscularly), and anesthesia was induced using a combination of ketamine (5 mg/kg intravenous) and diazepam (0.25 mg/kg intravenous). The animals were intubated and maintained under isoflurane (1–2%) anesthesia. Saline (10 mL/kg/h) was substituted through an intravenous catheter. The fur of the Beagles' lower abdomen was removed, and disinfection was performed using iodophor. A midline incision was made in the abdomen followed by exposure of the subcutaneous fat tissue. Subcutaneous fat tissue was removed with sterile forceps and reserved. The adipose tissue was rinsed with PBS, minced into small pieces, with vigorous shaking at 800 rpm, and then incubated in a solution containing 0.2% collagenase type IA for 20 minutes at 37°C with shaking at 200 rpm. The suspension was filtered through a 70 *μ*m cell strainer, and the top lipid layer was removed and centrifuged at 1500 rpm for 8 min at room temperature. The remaining cells were suspended in 10 mL Dulbecco's modified Eagle medium (DMEM) supplemented with streptomycin, fungizone, penicillin, and 10% fetal bovine serum (FBS), plated at a density of 1 × 10^6^ cells in a 10 cm dish and cultured at 37°C in 5% CO_2_. After 24 hours, the cells were rinsed with PBS. Through the detection of specific surface antigens and the ability of multidirectional differentiation of cells, we determined that they were adipose-derived mesenchymal stem cells.

### 2.3. Scaffold Preparation and Cell Seeding

The prepared acellularized arterial matrix was incubated for 24 h in DMEM supplemented with 10% fetal bovine serum (FBS) prior to implantation. Third-generation adipose-derived mesenchymal stem cells were seeded onto the arterial matrix at a concentration of 5 × 10^6^ cells/cm^2^ and in a 15 mL centrifuge tube filled with DMEM/10% FBS. There was intermittent shaking in a 5% CO_2_, 37°C incubator with a rotary shaker; the medium was stirred at 10 rpm to allow for uniform distribution of cells over a 24 h period. It was shaken for 30 minutes and then stood still for 30 minutes. After repeating a total of 3 cycles, it was cultured for 24 hours. The coculture of the cell-scaffold complex was observed under a fluorescence microscope and confocal microscope.

### 2.4. Surgical Procedures

**Figure 1:**
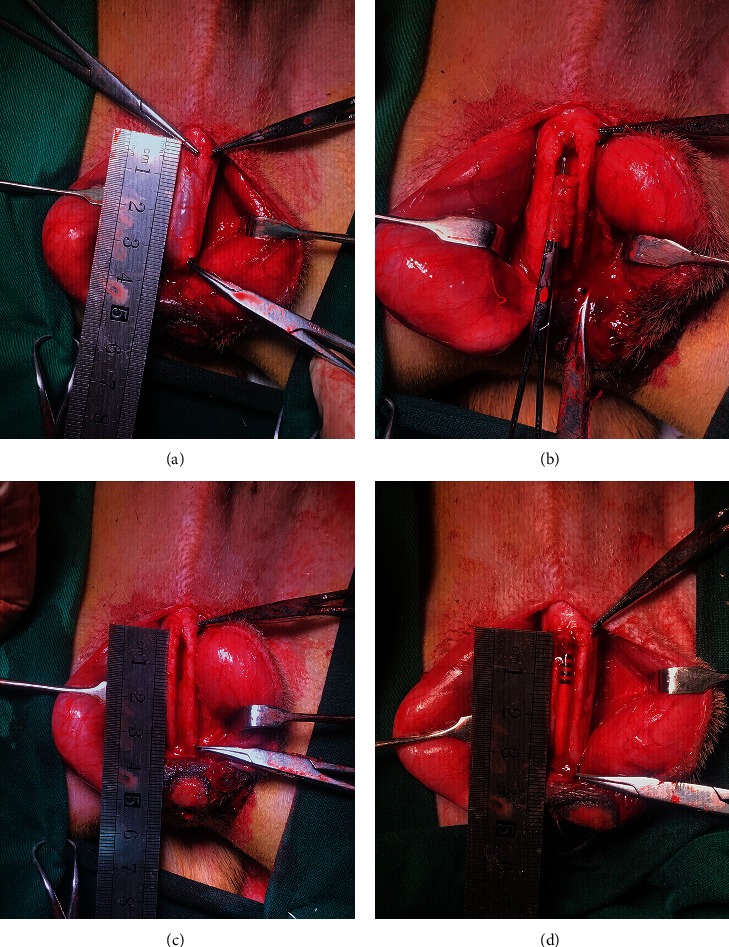
Surgical procedures of urethroplasty. A 3 cm long segment of the urethra (about 17% of the entire length of the urethra) starting distal to the bulbous urethra was transected and removed (a–c). The cell-seeded acellular arterial matrix was used to substitute the urethral defect (d).

A total of 12 animals were used in this study, in which 6 animals received cell-seeded acellular arterial matrix and the other 6 received unseeded. Under anesthesia, a suprapubic bladder catheter was surgically inserted into the bladder to ensure proper drainage of urine during healing and indwelling 6F catheter in the urethra to support the urethra for 2 wk. Subsequently, a median longitudinal incision of the perineum was taken, and the skin and subcutaneous tissue were separated to expose the urethra. The entire length of the urethra is about 18 cm, and a 3 cm long segment of the urethra (about 17% of the entire length of the urethra) starting distal to the bulbous urethra was transected and removed. The cell-seeded acellular arterial matrix was interposed, and the ends were anastomosed with the native ends of urethral tissues using 5-0 vicryl sutures in the experimental group (Figure 1), while the acellular arterial matrix was used to substitute the urethral defect in the control group. The wound was closed in layers in a routine fashion. All animals were subcutaneously injected with meloxicam for pain and subcutaneously injected with enrofloxacin 0.1 mL/kg for anti-inflammatory symptomatic until the catheter was removed.

### 2.5. Postoperative Assessment

The animals (*n* = 12) were divided over two time points. Retrograde urethrograms and graft harvest were performed at 1 and 3 months after implantation. Animals were euthanized using the anesthetic drug, and the entire urethra was circumscribed with sharp dissection. The nonabsorbable sutures were identified to demarcate the graft margins. Sections of the graft were obtained for histological and immunohistochemical analyses. All tissues were formalin-fixed, paraffin-embedded, and sectioned (8 mm). Hematoxylin and eosin and Masson's trichrome staining was performed. Immunohistochemical staining was performed using monoclonal antibodies against the urothelial cell layer (pancytokeratins AE1/AE3), smooth muscle cell layer (*α*-SMA), and CD34.

## 3. Results

### 3.1. Cell-Seeded Acellular Arterial Matrix

**Figure 2:**
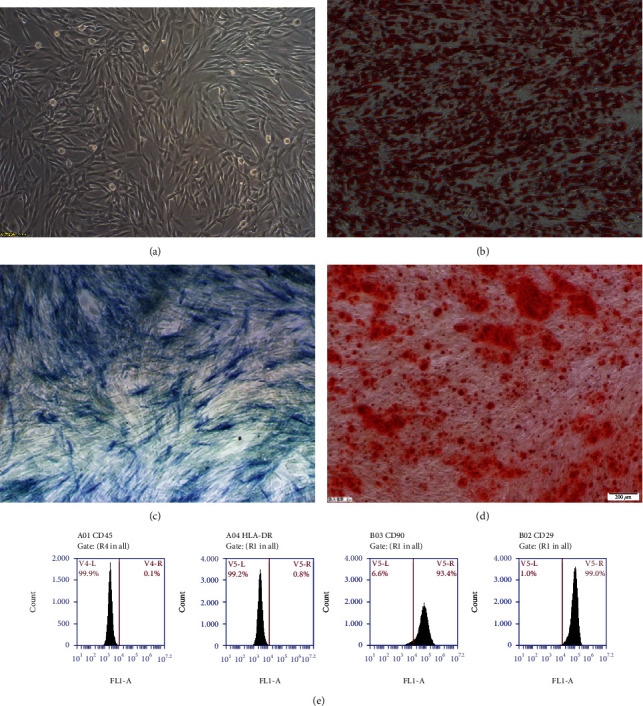
Morphology and identification of ADSCs. (a) Morphology of ADSCs under a light microscope. (b) Oil Red O staining results on the 11th day of adipogenic differentiation. (c) ALP staining results on the 6th day of osteogenic differentiation. (d) Alizarin Red staining results on the 12th day of osteogenic differentiation. (e) Identify its phenotype by flow cytometry.

Cells obtained from canine abdominal subcutaneous adipose tissue were characterized by their ability to express stem cell markers and to differentiate toward adipogenic and osteogenic lineages when cultured in media containing lineage-specific factors. The known MSC markers such as CD29 and CD90 were highly expressed by the cells. Negative markers such as CD45 and HLA-DR were not expressed (Figure 2(e)). The multilineage plasticity of ADSCs was confirmed by specific staining methods: Oil Red O staining and Alizarin Red S staining, respectively (Figures 2(a)–2(d)).

**Figure 3:**
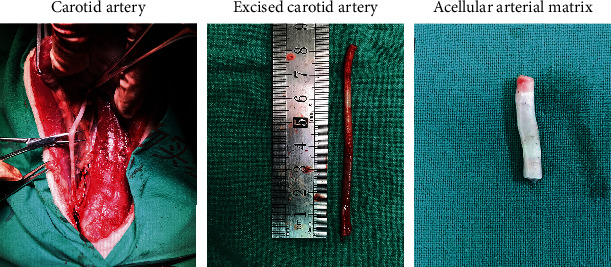
Preparation of the acellular arterial matrix. Carotid arteries were obtained by surgical resection from donor animal and decellularized.

**Figure 4:**
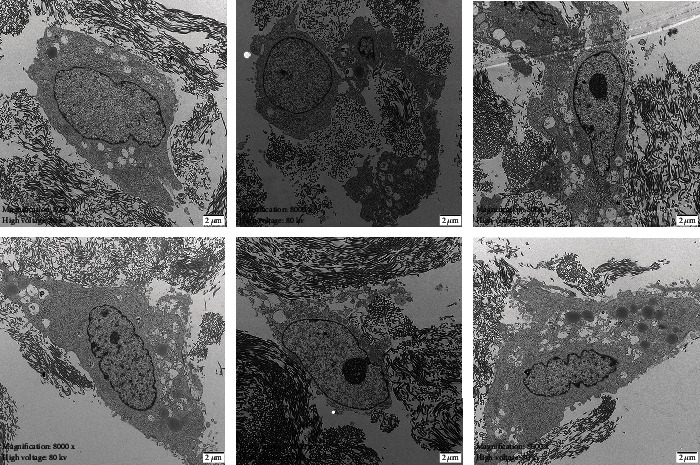
The cell-scaffold complex was observed under a transmission electron microscope.

Histological analyses of the in vitro processed carotid arterial matrices confirmed their acellularity. After mechanical and chemical treatments, the acellular arterial matrix became translucent membranes and was milky white exhibiting elasticity and toughness (Figure 3). The ADSCs were cocultured with an acellular arterial matrix for 24 h, and then, these cells were observed under a confocal microscope (Figure 4).

### 3.2. Surgical Outcomes

**Figure 5:**
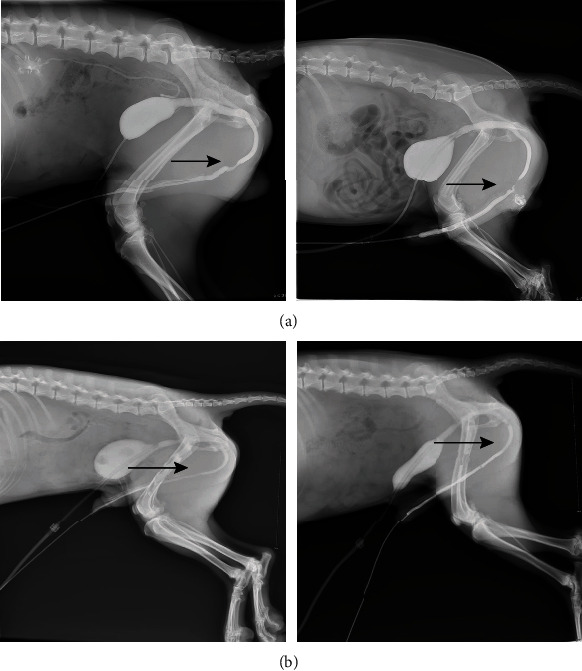
Postoperative urethrography. (a) Urethral stricture and urine leakage were confirmed in the control group. (b) Wide urethral calibers were demonstrated in the experimental group.

**Figure 6:**
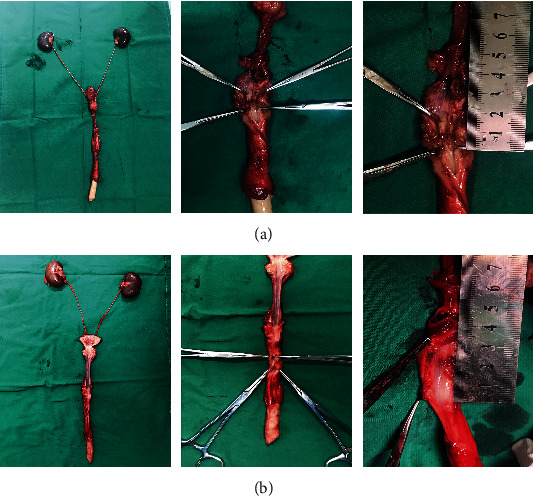
Gross examination of the reconstructed urethra. Graft contracture and scar formation were showed in the control group (a). The graft was highly epithelialized and smooth in the experimental group (b).

**Figure 7:**
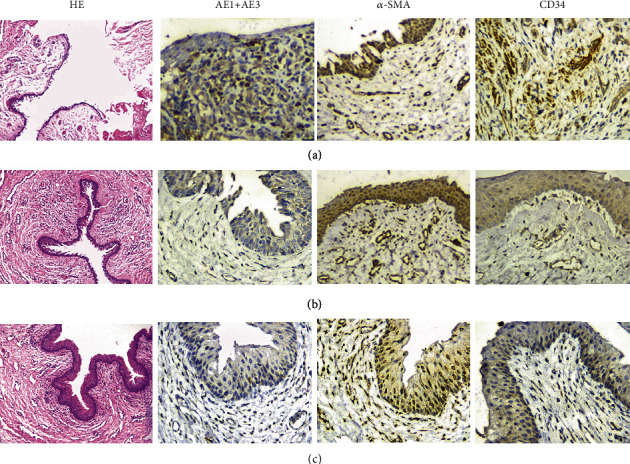
Histological analysis of reconstructed urethra: (a) the control group; (b) the experimental group; (c) normal urethral tissue (HE ×100, immunohistochemistry ×400).

Two animals in the control group implanted with acellular matrices had early urine leakage after removal of the urethral catheter, which was due to fistula formation at the anastomotic site. The urethral stricture was demonstrated in 3 animals in the control group after 3 mo of implantation as shown via the urethrography (Figure 5(a)). In contrast, serial urethrography confirmed maintenance of a wide urethral caliber without any signs of strictures in 6 animals implanted with cell-seeded tubularized matrices (Figure 5(b)). The region of the grafts was clearly delineated and identified by nonabsorbable sutures. Gross examination of the cell-seeded arterial matrix showed that the graft was highly epithelialized and smooth (Figure 6(b)), while graft contracture and scar formation were showed in the control group (Figure 6(a)). Histologic analysis of the cell-seeded arterial matrix at 1 month confirmed the presence of multilayered urothelium and muscle. The levels of tissue formation developed over time with a progressive increase in muscle content (Figure 7(b)). In contrast, extensive fibrosis and sparse smooth muscle were seen in animals treated with the matrix without cell (Figure 7(a)). Using anti-CD-34 antibodies, angiogenesis was noted on the cell-seeded scaffolds within the organizing seromuscular layers (Figure 7(a)). However, the unseeded grafts demonstrated a scarcity of vascular organization in comparison to their seeded counterparts (Figure 7(a)).

## 4. Discussion

Extensive urethral reconstruction is often recommended in a variety of urethral conditions, including inflammatory and posttraumatic strictures, congenital defects, and malignancy [21–23]. Most of the grafts used in urethral reconstruction are derived from autologous tissues, such as genital and extragenital skin flaps, bladder and buccal mucosa, tunica vaginalis, and bowel mucosa [24–27]. Because of its high success rate, buccal mucosa remains the most widely used source of tissue for urethral replacement, especially in cases of complex urethral reconstruction [28, 29]. However, donor site complications have been reported associated with the buccal mucosa harvesting, such as oral pain, swelling, salivary tube injury, speaking disorders, oral tightness, dysgeusia, and scar deformities. This limits the application of autologous tissues in urethral repair [30].

Tissue engineering and regenerative medicine studies have led to the development of various biomaterial scaffolds that can be used for urethral repair, such as synthetic materials, collagen, and acellular matrix [15, 17, 31, 32]. Dorin et al. proved that acellular matrices can be successfully used in tubularized urethral reconstruction, but the maximum potential distance of normal native tissue regeneration was limited when using tubularized unseeded matrices [33]. Results from the study of De Filippo et al. and Orabi et al. demonstrated that collagen scaffolds seeded with cells can be used for long tubularized urethral replacement, whereas scaffolds without cells lead to poor tissue development and strictures. These results confirmed the importance of seeded cells in urethral tissue engineering [34, 35].

Experimental results in this study show that tissue-engineered acellular arterial matrix seeded with ADSCs can be successfully used for urethral replacement. From the subcutaneous fat tissue, we were able to isolate ADSCs, which were characterized by flow cytometry analysis for positive expression of CD29, CD90, and CD44 and negative expression of CD45. Then, we expanded ADSCs until sufficient numbers were reached and labeled them with a fluorescent marker before scaffold seeding. After discontinuous shaking coculture, the ADSCs lived well and presented a good cell adhesion in the ADSC-scaffold complex, which were confirmed by the fluorescence microscope and confocal microscope.

The ADSC-seeded arterial matrix appears to function as a scaffold to remodel and regenerate a near-normal dog urethra. The animals receiving the ADSC-seeded arterial matrix showed improvement over the control group in all histologic and functional aspects. We demonstrate that a multilayered columnar epithelium was regenerated on the luminal side of the scaffold while a complete muscle layer was formed on the corresponding outer surface. All animals had a wide urethral caliber without any signs of strictures in the experimental group. In comparison, the control animals developed at most a single layer of stratified epithelium postoperatively and 3 animals had urethral strictures. In addition, fistula formation and early urine leakage were noted in 2 animals in the control group.

The reasons why ADSCs can reduce the occurrence of urethral stricture are as follows: (1) rapid development of the epithelium along the luminal surface of the urethra serves as a barrier that may prevent urine leakage into the suburothelial tissue and associated fibrosis, (2) the rapidly developing muscle cells differentiated from the ADSCs give the support for the urethral regeneration and keep the urethra from collapsing [36, 37], (3) ADSCs also provoke paracrine iNOS expression to exert antioxidative effects and to augment microhemodynamics that contributes to tissue repair and antifibrotic actions [38, 39], and (4) ADSCs may decrease the expression of fibrosis-associated genes and counteract urethral stricture formation [40, 41]. However, these mechanisms would not be present in the unseeded arterial matrix.

This study provides evidence that the ADSC-seeded tubularized arterial matrix can be used in the reconstruction of long urethral defects. However, there were still several limitations to this study. First, the current study was performed in the model with a normal healthy urethra, which could not fully simulate the fibrotic urethra bed in the clinical situation. Second, the number of experimental animals that were evaluated per time point is small. Larger sample sizes are needed to verify these results. Finally, the follow-up duration is only 3 months, which may not be able to catch all graft failures or developments of strictures. Therefore, further investigation in a larger series with a longer follow-up is required before introducing this technology into clinical practice.

## 5. Conclusions

This study provides preclinical evidence that the ADSC-seeded arterial matrix can be used as a tubularized scaffold in the reconstruction of a 3 cm long urethral defect in a male canine model. The ADSC-seeded arterial matrix remodels and regenerates normal-appearing urethral tissue layers over time. However, its clinical application will always be very limited, and more studies are required before introducing this technology into clinical practice.

## Data Availability

The datasets generated during and/or analyzed during the current study are available from the corresponding authors on reasonable request.

## Additional Points

*Statement of Significance*. This study provides preclinical evidence that a ADSC-seeded arterial matrix can be used as a tubularized scaffold in the reconstruction of 3 cm long urethral defect in a male canine model. ADSC-seeded arterial matrix remodels and regenerates normal-appearing urethral tissue layers over time.
